# Newly designed curcumin-loaded hybrid nanoparticles: a multifunctional strategy for combating oxidative stress, inflammation, and infections to accelerate wound healing and tissue regeneration

**DOI:** 10.1186/s12896-025-00989-z

**Published:** 2025-06-19

**Authors:** Heidi M. Abdel-Mageed, Nermeen Z. AbuelEzz, Ahmed A. Ali, Amira Emad Abdelaziz, Dina Nada, Sahar M. Abdelraouf, Shahinaze A. Fouad, Abeer Bishr, Rasha A. Radwan

**Affiliations:** 1 Molecular Biology Department, National Research Centre, Dokki, Giza, Egypt; 2 Biochemistry Department, College of Pharmaceutical Sciences and Drug Manufacturing, Misr University for Science and Technology, Cairo, Egypt; 3 Pharmacology Department, Clinical and Biological Sciences Departments, College of Pharmacy, Arab Academy for Science and Technology and Maritime Transport, Abu Kir, Alexandria, Egypt; 4 Pharmacology and Biochemistry Department, Faculty of Pharmacy, The British University in Egypt (BUE), Cairo, Egypt; 5 Department of Biochemistry, Faculty of Pharmacy, Misr International University (MIU), Cairo, Egypt; 6 Department of Pharmaceutics and Pharmaceutical Technology, Faculty of Pharmacy, Ahram Canadian University, Giza, Egypt; 7 Pharmacology and Toxicology Department, Faculty of Pharmacy, Ahram Canadian University, Giza, Egypt; 8 Biochemistry Department, Faculty of Biotechnology, German International University, Regional Ring Rd, East Cairo, New Administrative Capital, Egypt

**Keywords:** Curcumin, Hydrogels, Wound healing, Antioxidant anti-inflammatory, Hybrid nanoparticles, Cyclodextrin, Tissue regeneration

## Abstract

Effective treatment of skin wounds remains a clinical challenge owing to factors such as microbial infections, impaired fibroblast activity, disrupted angiogenesis, and collagen remodeling. In this study, we developed and evaluated novel curcumin–cyclodextrin hybrid nanoparticles (Cur/CD-HNPs) as a multifunctional platform for enhanced wound healing. Nanoparticles (NPs) were prepared via nanoprecipitation. Physicochemical and structural properties were systematically characterized by determining the encapsulation efficiency (EE), particle size, zeta potential, X-XRD, FTIR, SEM, in vitro release, and stability studies. The optimized Cur/CD-HNPs demonstrated a uniform particle size of 150.5 ± 2.8 nm, a surface charge of − 18.5 ± 0.59 mV, a PDI of 0.20 ± 0.03, and a high EE (90.2 ± 2.35%). Cur/CD-HNPs exhibited potent anti-inflammatory effects (97.93 ± 1.24% inhibition of protein denaturation), full antioxidant activity (100% ABTS radical scavenging, IC50 = 12.85 µg/mL), and broad-spectrum antibacterial efficacy. Cur/CD-HNPs exhibited a sustained biphasic release profile, with ~ 82% of Cur released over 24 h, supporting sustained delivery for wound healing applications. In vitro scratch assays revealed enhanced fibroblast proliferation and migration. For in vivo evaluation, the nanoparticles were incorporated into a hydrogel base and applied topically in a rat burn wound model, resulting in significantly accelerated wound closure (*P* < 0.05). Histopathological examination revealed improved epithelialization, collagen deposition, and tissue regeneration compared with the control groups. Our findings presented Cur/CD-HNPs as a promising therapeutic approach, offering Cur enhanced bioactivity, stability, and regenerative potential. This formulation addresses the key limitations of curcumin and presents a multifunctional and strong translational platform for clinical wound care.

**Graphical Abstract:**

**Figure:**
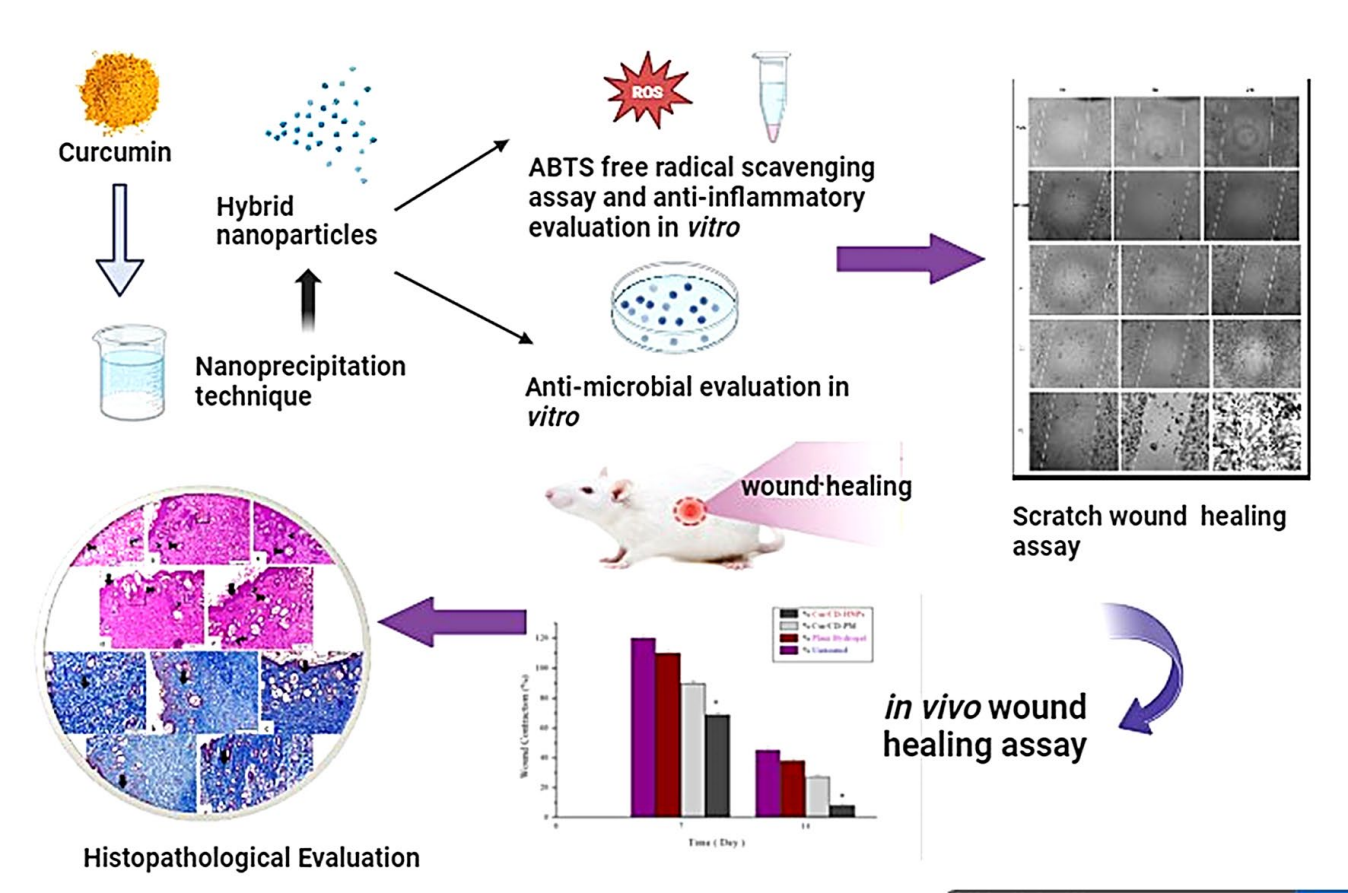

**Supplementary Information:**

The online version contains supplementary material available at 10.1186/s12896-025-00989-z.

## Introduction

Dermal injuries, including burns, surgical incisions, and trauma, remain a highly prevalent and socioeconomically stressful concern in the global healthcare system. Approximately 11 million individuals suffer from serious burns annually worldwide, necessitating considerable medical care [1]. Research indicates that over 15% of wounds fail to heal one year after onset, which frequently causes patients, their families, and healthcare systems to bear unnecessary and extended burdens [2, 3]. Chronic wounds, unlike acute wounds, are worsened by prolonged inflammation and factors like age, obesity, trauma, diabetes, and cancer, often resulting in scarring [4]. Wound healing involves an intricate interplay between hemostasis, inflammation, proliferation, and tissue remodeling. The disruption of this complex route could cause wound healing failure, resulting in non-healing wounds [5, 6]. Therefore, effective wound care remains a serious challenge.

Curcumin (Cur) is a highly ranked medicinal plant for wound healing. It is a natural, low-molecular-weight hydrophobic polyphenolic compound identified in *Curcuma aromatica* and *Curcuma longa*. Antioxidant, anti-inflammatory, antimicrobial, and anticarcinogenic properties were reported among Cur’s multifunctional attributes [7]. It has been used for several inflammatory diseases and cancers, owing to its wide spectrum of biological and pharmacological properties [8]. Applying Cur topically, as opposed to orally or systemically, allows it to reach the wounded area more efficiently, which speeds up the healing process of cutaneous wounds. Cur promotes the synthesis of growth factors, such as VEGF, FGF, EGF, and TGF-β. Moreover, it protects against oxidative tissue damage, accelerates re-epithelialization, and enhances tissue granulation and vascularization [9]. Additionally, Cur facilitates a swift transition from the inflammatory to proliferative stages of healing, which is distinguished by increased collagen deposition, accelerated re-epithelialization, enhanced neovascularization, and tissue development [9]. However, low solubility, low stability, photosensitivity, poor absorption, and poor water solubility (0.4 µg mL − 1) in physiological media impair its application [10]. Hence, the creation of Cur formulations has been the focus of extensive research using a variety of procedures and methods.

To overcome curcumin’s poor solubility and stability, host-guest complexes, particularly with cyclodextrins (CDs), are widely used [11]. β-Cyclodextrin (β-CD), a cyclic oligosaccharide, is the most common host, offering advantages such as low cost, biocompatibility, and a size-appropriate hydrophobic cavity ideal for encapsulating Cur [12]. The primary characteristic of β-CD is its size-defined three-dimensional microenvironmental chiral chamber with hydrophilic and hydrophobic outer and inner rings, respectively. Its unique, tapered, conical structure enhances Cur’s water solubility and stability [11]. However, β-CD/Cur complexes alone face limitations, including uncontrolled release, systemic loss, and large particle size, which restrict their topical application. To enable localized, sustained release, especially for wounds, β-CD/Cur complexes can be embedded into nanoscale carrier systems for targeted delivery [13].

Nano formulations revolutionize curcumin’s disease management by enhancing its solubility, bioavailability, and targeted delivery. The increased surface area of nano-curcumin exposes more hydroxyl groups, facilitating strong hydrogen bonding with the water molecules, significantly improving its absorption and systemic distribution. This allows lower doses to achieve higher therapeutic efficacy while minimizing systemic toxicity and off-target effects. Beyond delivery, nano-curcumin actively modulates the pathological microenvironment, reducing disease progression, recurrence, and metastasis, positioning it as a next-generation therapeutic with broad clinical potential [14–16]. Hybrid nanoparticles (HNPs), combining the strengths of inorganic and lipid nanoparticles, offer a powerful platform for delivering both hydrophilic and hydrophobic compounds [5, 17]. They addressed key limitations of conventional systems, such as lipid nanoparticle instability, payload leakage, and polymer biocompatibility issues [18, 19]. HNPs enable controlled, long-term release, targeted delivery, and versatile architectures, making them suitable for synergistic therapies and theranostic applications [20]. Thus, HNPs present a superior alternative to traditional nanoparticles, enhancing Cur delivery with improved stability, efficacy, and therapeutic outcomes.

Curcumin-loaded hybrid Nanoparticles (Cur/CD-HNPs) offer a significant advancement over various nanoparticle-based drug delivery systems, particularly for applications in wound healing. The unique properties of HSA-based nanoparticles, such as their biocompatibility, stability, and ability to enhance Cur’s solubility, make them ideal for promoting tissue regeneration and accelerating wound healing. Compared to liposomes, which are prone to instability due to lipid oxidation and premature drug release, Cur/CD-HNPs provide a more controlled release of Cur, ensuring sustained therapeutic effects without compromising the healing process [21]. polylactic-co-glycolic acid (PLGA)-based nanoparticles, commonly used for wound healing applications, also offer controlled drug release but are often limited by their relatively slower degradation rate and the potential for inflammatory responses due to the accumulation of polymeric residues [22, 23]. In contrast, the ability of Cur/CD-HNPs to enhance cellular uptake and their biocompatible nature allow them to effectively target cells involved in the wound healing process, such as fibroblasts and keratinocytes, promoting tissue regeneration without triggering significant immune responses [20, 24]. Other nanoparticle types, such as chitosan-based nanoparticles and dendrimers, have also been explored for wound healing due to their antimicrobial properties and ability to form gels for local drug delivery. However, chitosan nanoparticles may not offer as high a drug loading capacity or sustained release as Cur/CD-HNPs, potentially limiting their efficacy in long-term wound management [25]. While highly effective in targeting specific cell receptors and delivering high drug concentrations, Dendrimers can exhibit cytotoxicity at higher concentrations, which may hinder their clinical application in wound healing [23]. In contrast, Cur/CD-HNPs maintain a balance of high Cur loading, targeted delivery, and minimal toxicity, making them a promising formulation for wound healing, where prolonged release and minimal irritation are crucial for effective recovery [26].

Cyclodextrin inclusion complexes have emerged as effective carriers, but their integration into hybrid nanoparticle (HNP) systems remains largely underexplored. To date, our review of the literature has identified only one report describing β-CD/Cur inclusion complexes formulated via nanoprecipitation, indicating a striking gap in both formulation science and application-oriented research. The feasibility of NPs synthesis using the nanoprecipitation method has been reported in various studies [27–29]. The excellent binding characteristics of CD toward guest molecules when incorporated into NPs offer an intriguing blend of nanotechnology and supramolecular and macromolecular chemistry [30].

This study directly addresses this gap by introducing a novel, multifunctional Cur/CD-based hybrid nanoparticle (Cur/CD-HNP) system designed to overcome the critical limitations of Cur delivery and unlock its full therapeutic potential in wound healing. Compared with conventional carriers, such as emulsions or liposomes, HNPs have been effectively used for delivering lipophilic drugs, exhibiting enhanced biocompatibility [31, 32]. In this study, a tailored nanoprecipitation approach was employed to co-assemble β-CD, Pluronic F-86, and cholesterol into a stable, newly developed biocompatible nanoparticle matrix. Cholesterol, in particular, plays a pivotal role in enhancing membrane rigidity, promoting self-assembly, and reducing drug leakage, collectively boosting nanoparticle stability and drug-loading capacity [31, 32]. To fully understand the therapeutic superiority of nano-curcumin over native Cur, a comparative study is essential. Beyond formulation, this study comprehensively characterized the developed HNPs in terms of their physicochemical attributes, antioxidant capacity, antibacterial efficacy, and anti-inflammatory properties. Importantly, we also evaluated their biological performance through in vitro assays for cytocompatibility, cell proliferation, and migration, followed by in vivo wound healing and histological analyses. This study presents a novel, multifunctional nanoparticle system that addresses a persistent gap in the literature and offers a promising strategy for advancing curcumin-based therapeutics in wound management. In summary, this work delivers a first-of-its-kind hybrid nanosystem that advances Cur delivery science and also offers a robust translational pathway for accelerating wound repair, bridging a major gap between curcumin’s therapeutic promise and clinical application.

## Materials and methods

### Materials

Curcumin (Cur), β-cyclodextrin (CD), pluronic-86, Carbopol, and cholesterol were purchased from Sigma-Aldrich and used without additional purification. Dulbecco’s modified Eagle’s medium (DMEM), fetal bovine serum (FBS), L-Glutamine, and Penicillin/Streptomycin were purchased from Lonza Bioscience. All solvents employed for spectrophotometric investigations were of spectrophotometric quality. Solubilization and other chemical studies were conducted using Milli-Q water. All solvents used in this study were of analytical grade.

### Preparation of β–cyclodextrin–curcumin hybrid nanoparticles (Cur/CD-HNPs) by nanoprecipitation

**Fig. 1:**
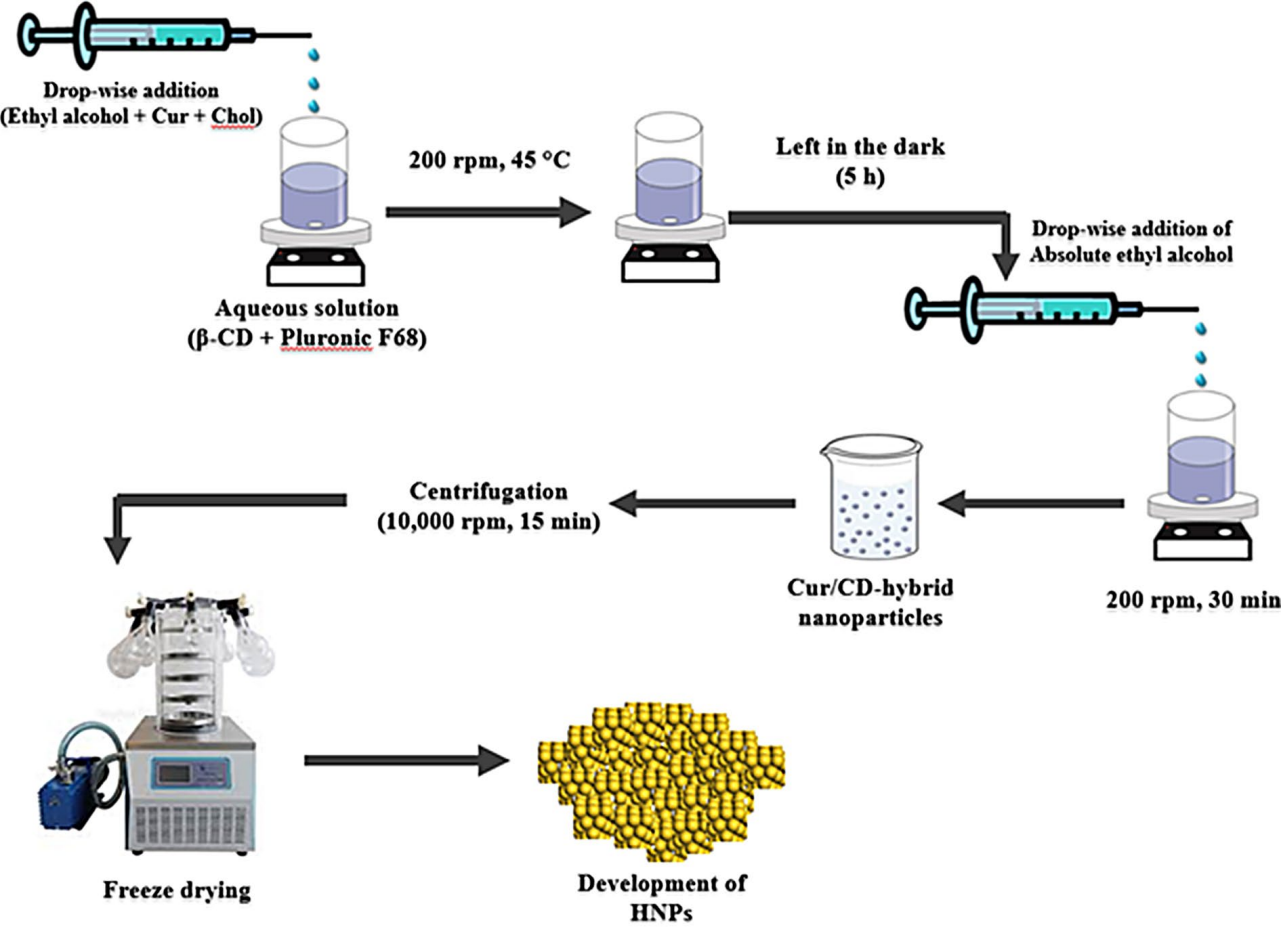
Schematic presentation of nanoprecipitation technique

A modified nanoprecipitation method was used to prepare Cur/CD-HNPs [27, 28, 33] using a mixture of cholesterol and Pluronic F-68 as formulation components and a molar ratio of Cur to β-CD of 1:1 [29, 34]. In brief, a β-CD aqueous solution (10 mM) was prepared by dissolving 113.5 mg of β-CD in 10 mL of water and 0.5% (w/v) Pluronic-F68. Next, 10 mL of 100% ethanol containing 368 mg Cur and cholesterol (193.5 mg) was prepared. 1 mL of this mixture was added dropwise to the β-CD aqueous solution under continuous stirring (200 rpm, 45 °C). The resulting mixture was constantly mixed in the dark for five hours. Thereafter, the nanoprecipitation technique was used to create the Cur/CD-HNPs. Using a syringe pump and stirring, 66 mL of absolute ethanol was added dropwise at 1.5 mL/min to the Cur/CD-HNPs solution as a nonsolvent. The mixture was then magnetically stirred at 200 rpm for 30 min. The Cur/CD-HNPs were spontaneously generated and recovered by centrifugation (10000 rpm for 15 min, at 5 ^o^C). It was concentrated and freeze-dried to obtain HNP, which was stored at 4 °C until use, as shown in Fig. 1. Unloaded HNPs were prepared for comparison and named as Free HNPs. (Supplementary Table S1). All procedures for the Cur-HNPs were completed in the dark.

### **Physical mixture of cur/** β-**CD**

A homogenous physical mixture (PM) was created by mixing β-CD (3.41 g) and Cur (0.87 g) in a mortar using a plastic spoon. This mixture served as the control group and was designated as Cur/CD-PM.

### Preparation of the cur-based hydrogel matrix

To achieve system gelation, 20% w/v of either Cur/CD-PM or Cur/CD-HNPs was dispersed in 25 mL phosphate buffer saline (pH 7.4), followed by the addition of carbopol at a concentration of 2% w/w while gently stirring the mixture with a glass rod. Triethanolamine was added to ensure the gelation of the system. This process ensured complete drug loading into the hydrogel matrix. A blank carbopol gel was synthesized at an equivalent concentration; for simplicity, this preparation is referred to as a plain hydrogel.

### Hybrid nanoparticles characterization

#### Determination of the drug encapsulation efficiency (EE)

Centrifugation was used to collect HNPs and determine the encapsulation efficiency (EE%) of Cur inside the HNPs [35, 36]. The Initial UV–Vis spectra of Cur in DMSO were measured using a V-650 UV–Vis spectrophotometer (JASCO International Co., Ltd., Tokyo, Japan). The absorption peak at 420 nm was chosen to create a calibration curve and to assess the EE% of the Cur inside the Cur/CD-HNPs. The calibration curve was produced for Cur concentrations in the range 0.5 and 8 µg/mL, with correlation analysis leading to R^2^ = 0.9999, using Microsoft Excel (Redmond, Washington, USA). The newly generated Cur/CD-HNPs were separated from the Free Cur by centrifugation at 10,000 rpm for 30 min at 4 °C to collect the supernatants. Alcohol was used to dilute the supernatants such that the highest Cur concentration was 8 µg/mL. The validity of the method was confirmed by measuring the amount of Cur at 425 nm in both HNPs and free Cur in the supernatant. The drug entrapment efficiency (EE%) and drug-loading capacity (DL%) were determined using the following equations [37]:

1 $$\:EE\%=\raisebox{1ex}{$\text{T}\text{o}\text{t}\text{a}\text{l}\:\text{C}\text{u}\text{r}-\text{F}\text{r}\text{e}\text{e}\:\text{C}\text{u}\text{r}$}\!\left/\:\!\raisebox{-1ex}{$\text{T}\text{o}\text{t}\text{a}\text{l}\:\text{C}\text{u}\text{r}$}\right.\times\:100$$

2 $$DL\% = {\raise0.7ex\hbox{$\begin{gathered}{\text{Weight}}\,{\text{of}}\,{\text{Cur}} \hfill \\{\text{in}}\,{\text{nanoparticles}} \hfill \\ \end{gathered} $} \!\mathord{\left/{\vphantom {\begin{gathered}{\text{Weight}}\,{\text{of}}\,{\text{Cur}} \hfill \\{\text{in}}\,{\text{nanoparticles}} \hfill \\ \end{gathered} \begin{gathered}{\text{Total}}\,{\text{weight}}\,{\text{of}} \hfill \\{\text{nanoparticles}} \hfill \\ \end{gathered} }}\right.\kern-\nulldelimiterspace}\!\lower0.7ex\hbox{$\begin{gathered}{\text{Total}}\,{\text{weight}}\,{\text{of}} \hfill \\{\text{nanoparticles}} \hfill \\ \end{gathered} $}} \times 100$$

#### Particle size and zeta-potential measurements

The size distribution (polydispersity index, PDI), surface charge (zeta potential, Z-pot), and mean particle size (hydrodynamic diameter) of the HNPs were measured using a Malvern Zetasizer Nano (Malvern Instruments Ltd., Malvern, UK). Three sets of measurements were performed at 25 °C, using a medium refractive index of 1.33 and a viscosity of 0.8872 cP. To prevent multiple scattering, the samples were diluted 100 times using double-distilled water before analysis [38]. The dynamic light scattering (DS) and polydispersity index (PDI) were then determined by averaging 13 measurements of each set at a backscattering angle of 173°. After the particle size measurements, the potential was measured in the same cuvette, taking three consecutive readings for each sample. The mean value and standard deviation were then calculated.

#### Fourier transform infrared (FTIR) spectroscopy

FTIR analysis of the Cur, CD, Cur/CD-PM, and Cur/CD-HNPs was conducted using an FTIR spectrometer (PerkinElmer FTIR-1600, USA) in the wavenumber range 400–4000 cm^− 1^ at a resolution of 4 cm^− 1^, as previously described [34, 39, 40].

#### X-ray diffraction (XRD) analysis

The X-ray diffraction patterns of the Cur, CD, Cur/CD-PM, and Cur/CD-HNPs were obtained using a PAN analytical X-ray diffraction apparatus (model X’ Pert PRO) equipped with a Secondary Monochromator, utilizing Cu radiation (λ = 1.542 Å) at 45 kV and 35 mA and a scanning speed of 0.04°/s. The samples were analyzed using a monochromatic Cu-Kα radiation source in the step-scan mode with a 2θ angle varying from 2° to 60°. The method is presented in previous studies [39–41].

#### Scanning electron microscope (SEM) morphology analysis

The surface morphology of the Cur/CD-HNPs was analyzed using scanning electron microscopy (SEM) with a Quanta 250 FEG (Thermo Fisher Scientific, USA) at the National Research Centre (Cairo, Egypt). Before imaging, the samples were sputter-coated with a thin layer of gold using a Jeol JSM-6510 coater (JEOL Ltd., Tokyo, Japan) for 60 s to enhance the conductivity. SEM images were acquired at an accelerating voltage of 20.00 kV with a working distance (WD) of 12.7 mm. A backscattered electron detector (BSED) was employed, and the images were captured at a magnification of 4000×. The horizontal field width (HFW) was 104 μm, providing high-resolution visualization of the nano-powder particles’ spherical morphology and uniform distribution [42].

### Stability studies of CUR/β-CD nanoparticles

#### Evaluation of the pH stability

The effect of pH on the stability of free Cur, Cur/CD-PM, and Cur/CD-HNP was assessed using a previously reported procedure [43] with minor adjustments. Different pH conditions (4.0–10.0) were used to test the pH stability. NaOH and HCl were added to PBS, and the pH was adjusted to create a mixture. Following the addition of the samples, the mixtures were agitated for 180 min at 37 °C. The Cur retention rate (%) was determined by measuring the absorbance at 420 nm, using Eq. 3:

3 $$\begin{gathered}\:{\text{Cur}}\:{\text{Retention}}\:{\text{rate}}\:\left( {{\text{RR}}\% } \right) \hfill \\= \frac{{{\text{Remaining}}\:{\text{amount}}\:{\text{of}}\:{\text{Cur}}\:{\text{after}}\:{\text{treatment}}}}{{{\text{Initial}}\:{\text{amount}}\:{\text{of}}\:{\text{Cur}}}}\: \times \:100 \hfill \\ \end{gathered} $$.

#### Determination of the thermal stability

The samples were then subjected to a thermal treatment in an oven at 100 °C for 120 min [43]. Subsequently, the remaining Cur content was assessed by measuring absorbance at 420 nm using Eq. 3.

#### Evaluation of long-term stability

Freeze-dried Cur/CD-HNP samples were kept at room temperature in a dark box (25 °C and 60% relative humidity (RH)) for three months to examine their long-term storage stability. The Cur (EE%) and physical stability of the HNPs were assessed by measuring their size and polydispersity. at defined time intervals (0, 15, 30, 60, and 90 days) during storage.

### ABTS free radical scavenging assay

ABTS (2,2’-azino-bis (3-ethylbenzo-thiazoline-6-sulfonic acid) radical scavenging activity was measured spectrophotometrically using the procedure outlined in an earlier study [7, 44]. Briefly, ABTS^•+^ solution was diluted to an absorbance of 0.750 ± 0.025 at 734 nm in 0.1 M sodium phosphate buffer (pH 7.4). After 30 min, the absorbance of a solution comprising 20 µL Cur/CD-HNPs or Cur/CD-PM sample (0.2–1 µg) and 1 mL ABTS^•+^ working solution was monitored at 734 nm. The free radical scavenging percentage was compared to the blank without a scavenger.

Free radical scavenging activity (%) was determined using Eq. 4:

4 $$ABTS\,scavenging\,ability\left( \% \right) = {\raise0.7ex\hbox{${At - Ab}$} \!\mathord{\left/{\vphantom {{At - Ab} {Ac}}}\right.\kern-\nulldelimiterspace}\!\lower0.7ex\hbox{${Ac}$}} \times 100$$

where At is the absorbance of the mixture of the samples and ABTS solution, Ab is the absorbance of deionized water, and Ac is the absorbance of the mixed solution.

### Anti-inflammatory activity assay

The Bovine serum albumin (BSA) denaturation assay was used to evaluate protein denaturation inhibition using salicylic acid as a standard, as described previously [45]. Briefly, the samples were dissolved in 100% ethanol to obtain a Cur concentration of 1 mg/mL. Various concentrations (125–1000 µg/mL) of the Cur solution were prepared using a 0.1 M phosphate buffer solution (pH 7.4). The reaction mixture (3 mL) contained 0.2 mL of 1% bovine albumin, 2.78 mL of buffer, and 0.02 mL of the Cur sample. The reaction mixture was incubated in a water bath for 15 min at 37 °C, and then heated for 5 min at 70 °C. After cooling the reaction mixture, the turbidity was recorded at 660 nm using a UV/VIS spectrometer (JASCO International Co., Ltd., Tokyo, Japan).

The percent inhibition of protein denaturation was determined using Eq. 5:

5 $$\begin{gathered}{\text{Denaturation}}\,{\text{Inhibition}}\left( {\text{\% }} \right) \hfill \\= {\raise0.7ex\hbox{$\begin{gathered}{\text{the}}\,{\text{Absorbance}}\,{\text{of}}\,{\text{control}} \hfill \\- {\text{Absorbance}}\,{\text{of}}\,{\text{test}}\,{\text{sample}} \hfill \\ \end{gathered} $} \!\mathord{\left/{\vphantom {\begin{gathered}{\text{the}}\,{\text{Absorbance}}\,{\text{of}}\,{\text{control}} \hfill \\- {\text{Absorbance}}\,{\text{of}}\,{\text{test}}\,{\text{sample}} \hfill \\ \end{gathered} {{\text{Absorbance}}\,{\text{of}}\,{\text{control}}}}}\right.\kern-\nulldelimiterspace}\!\lower0.7ex\hbox{${{\text{Absorbance}}\,{\text{of}}\,{\text{control}}}$}} \times 100 \hfill \\ \end{gathered} $$

### Anti-microbial efficiency evaluation

The disc diffusion method, as outlined previously [45, 46], was used to test the antimicrobial activity of the formulations against four bacterial strains: *Escherichia coli* (E. *coli*; ATCC 25923), Staphylococcus *aureus* (S. *aureus*; ATCC 8739), *Pseudomonas aeruginosa* (P. *aeruginosa*; ATCC 9027), and *Bacillus subtilis* (B. *subtilis*; ATCC 6633). In brief, 8 mm diameter filter paper discs (Whatman no. 1) were prepared and sterilized for the disc diffusion experiment. The discs were aseptically placed on nutrient agar plates seeded with the corresponding test microorganisms. These discs were filled with 100 µL of sample dispersions of either Cur/CD-HNPs or Cur/CD-PM, containing an equivalent volume of 50 µM Cur or free HNPs. The plates were incubated for 24 h at 37 °C. Following the ISO 20,645 methodology [47], clear inhibition zones around the tested samples were evaluated for antibacterial activity and measured for diameter (in mm). The control samples consisted of a plain hydrogel base and an untreated disk. To ascertain the antibacterial activity of the Cur/CD-HNPs as a function of time, the same process was performed after 21 days.

### In vitro release study of cur hybrid nanoparticles

In vitro release profile of Cur from the optimized Cur/CD-HNPs was assessed using the dialysis bag diffusion method [48]. A dialysis membrane with a molecular weight cut-off (MWCO) of 12–14 kDa was employed. The dialysis membrane was pre-treated by soaking in double-distilled water overnight. An accurately measured volume of the nanoparticle suspension (equivalent to 2 mg Cur) was placed into the pre-soaked dialysis bag, which was securely sealed and immersed in 100 mL phosphate-buffered saline (PBS, 0.01 M, pH 7.4). the dissolution medium contained 0.5% (v/v) Tween 80 to enhance the solubility of Cur and maintain the sink conditions. The experiments were performed at 37 ± 2 °C with continuous agitation at 50 rpm. At predetermined time intervals (1, 2, 4, 6, 8, 10, 12,16, 18, 20, 24, and 28 h), 5 mL aliquots of the release medium were withdrawn and replaced with an equal volume of fresh pre-warmed medium to maintain constant volume and sink conditions. Samples were filtered using a syringe filter (0.45 μm) and analyzed using a UV-Visible spectrophotometer at 420 nm after appropriate dilution. All experiments were conducted in triplicate, and the cumulative percentage of Cur released was calculated and plotted against time.

### Cytotoxicity assay

The tetrazolium salt 3-(4,5-dimethylthiazol-2-yl)-2,5-diphenyl tetrazolium bromide (MTT) assay was used to measure and evaluate the viability of primary human skin fibroblasts (HSF) treated with the formulated HNPs, as described previously [49]. Briefly, the cells were prepared and cultured in 96-well plates. The cells were grown in Dulbecco’s modified Eagle’s medium (DMEM) supplemented with 10% fetal bovine serum (FBS), 2 mM L-glutamine, 100 U/ml penicillin/streptomycin, 1 mM sodium pyruvate, and 0.1 mM non-essential amino acids at 37 °C in a humidified atmosphere (5% CO2 and 95% air) for 24 h. The next day, the cells were treated with various concentrations of T1 or T2 hydrogels (10, 5, and 2.5 mg/mL) in fresh complete DMEM and then incubated for 24 h at 37 °C and 5% CO_2_. After a 24 h incubation, 10 µL of MTT stock solution (5 mg/mL in phosphate-buffered saline, PBS) was added to each well and incubated for 3 h at 37 °C in a CO_2_ incubator. Following incubation, 100 µL of 100% dimethyl sulfoxide (DMSO) solubilization solution was added to each well and mixed well to dissolve the formed formazan crystals, which were then incubated for 15 min at 37 °C. The absorbance was measured at 570 nm using a Synergy HTX multimode plate reader (BioTek, Agilent). Untreated cells were used as a negative control, while 96% ethanol was used as a positive control because of its cytotoxic effects.

### Wound healing scratch assay

A wound healing assay was conducted to investigate the impact of formulated Cur/CD-PM (T1) and Cur/CD-HNPs (T2) on the migration of Human Skin Fibroblast (HSF) cells to determine their potential role in wound healing, according to a previously described method [50]. HSF cell lines were seeded in 24-well culture plates in complete Dulbecco’s modified Eagle’s medium (DMEM) supplemented with 10% fetal bovine serum (FBS), 2 mM L-glutamine, 100 U/mL penicillin/streptomycin, 1 mM sodium pyruvate, and 0.1 mM non-essential amino acids (NEAA). The cells were incubated at 37 °C, 5% CO_2_, and 95% relative humidity for 24 h to create a confluent monolayer. After the formation of the cell monolayer, a scratch-making procedure was performed. A sterile plastic micropipette tip was used to simulate the in vivo wound by creating a cell-free zone with a straight edge across the cell monolayer in each well. A gap width of 0.7 mm allows for observation at 20x magnification. After scratches were made, the wells were washed three times with phosphate-buffered saline PBS to remove any debris and detached cells, and a full medium was added. A test sample of the hydrogel at a final concentration of 10 mg/mL was added to the appropriate wells. Control samples were not exposed to the test samples (control group). The treatment was performed in low-serum DMEM (2%) for 24 h at 37 °C and 5% CO_2_. Scratch closure was monitored and visualized over time by capturing images of the scratch at different time points (0, 3, and 24 h) using an inverted microscope (Zoe Fluorescent Cell Imager, Bio-Rad, California, USA). Photographs for wound healing analysis were maintained under the following conditions. The cells were kept in a controlled environment at 37 °C with 5% CO_2_ to mimic physiological conditions during the imaging process. The cells were maintained in low-serum DMEM (2% FBS) to avoid interference with cell proliferation and wound closure. An inverted microscope was used with consistent 20x magnification to capture the photos. Photographs of the wound scratch were captured at the same spot as possible at specific intervals after wounding, immediately after the scratch, and at 3 and 24 h. Uniform bright-field illumination was maintained to ensure a clear visualization of the wound edges. To quantify the extent of scratch closure, images of the gap area were analyzed using ImageJ Software. The distance between scratch edges was measured to determine the degree of closure. Cell migration was expressed as a percentage of the initial migration observed at the zero-time point, which was considered to be 100%.

### Assessment of the in vivo wound healing efficiency

#### Burn wound experimental protocol

##### Animals

The wound healing characteristics of the Cur/CD-PM and Cur/CD-HNP-loaded hydrogel preparations were evaluated in a rat model. The study adhered to the Institute of Laboratory Animal Research’s “Guide for the Care and Use of Laboratory Animals” (Washington, DC, USA) and was approved by the Medical Ethics Committee of Cairo University (approval no: PT3519). The study was conducted following ARRIVE guidelines (https://arriveguidelines.org). Female Wistar rats (*N* = 32) weighing 150–200 g were obtained from the National Research Centre Laboratory Animal Resource Unit, Egypt. Animals were housed in polypropylene cages and maintained at 25 ± 5 ^o^C and 50 ± 5% humidity with 12-h light and dark cycles and had access to a standard diet and water ad libitum.

##### Establishment of skin burn wounds

Animals were anesthetized aseptically using ketamine (15 mg/kg) and xylocaine (1.1 mg/kg) intraperitoneally. A 1 cm^2^ stainless steel template was heated in boiling water for 5 min and applied to the shaved dorsal area for 10 s, causing partial-thickness burns [51].

##### Treatment of burn wounds

Animals were randomly assigned to four major groups (*n* = 8/group).

Group 1: untreated.

Group 2: treated with the plain hydrogel.

Group 3: group treated with the Cur/CD-PM loaded hydrogel matrix.

Group 4: group treated with the Cur/CD-HNPs loaded hydrogel matrix.

After wound establishment, 0.5 g of treatment (50 mg Cur/g) was applied directly and repeated twice daily to the wounded skin for 14 days. Each rat was caged separately to prevent biting and scratching, and each rat was separately caged.

##### Evaluation of the wound healing area

Digitized wound photographs were obtained on Days 1, 3, 7, and 14. Vernier calipers were used to measure the wound area at certain time points after treatment to assess wound closure.

The percentage of wound contraction/closure was determined by considering the initial wound size as 100% using the following formula (6) [31]:

6 $$\:\text{\%}\:\text{W}\text{o}\text{u}\text{n}\text{d}\:closure\:=\frac{A0-At}{A0}\:\times\:100$$

Where A0 is the original burn wound area, and At is the burn wound area after treatment at the time of observation.

#### Histopathological evaluation

##### Hematoxylin and Eosin (H&E) staining

Following the assigned group treatment, skin samples were taken at specified time intervals, embedded in paraffin, fixed in 10% formalin saline solution, and sliced into 4–6 μm-thick sections perpendicular to the wound surface. The corresponding skin sections were stained with hematoxylin and eosin (H&E) following a published methodology for histological examination. H&E-stained sections were examined using a Leica microscope (Leica Microsystems, Switzerland).

##### Masson’s trichrome staining

Masson’s trichrome was used to stain the tissue sections using the Masson’s trichrome staining kit (Sigma-Aldrich, St Louis, MO, USA) to enable the direct viewing of collagen fibers and histological evaluation of collagen deposition [52]. An optical microscope (Nikon Eclipse Ni-E, Japan) was used to capture images.

### Statistical analysis

The results of the triplicate experiments are presented as the mean ± standard deviation (± S.D). Two-way ANOVA was used, followed by Student’s t-test, to determine significant differences between sets of data. Statistical significance was set at *P* < 0.05. GraphPad Prism 6 (GraphPad Software, San Diego, California, USA) was used for all the statistical analyses.

## Results and discussion

### E**ncapsulation efficiency and cur loading capacity**

Curcumin’s sensitive nature, reduced water solubility, and low bioavailability restrict functional activity [53]. Studies have demonstrated that encapsulating Cur in a nanocarrier system can alleviate these limitations [29]. The nanoprecipitation technique was used to quickly and reliably create the HNPs. The precipitation process includes the continuous addition of a CD solution to a nonsolvent or, vice versa. The rapid formation of NPs is attributed to the interfacial turbulence between the solvent and non-solvent interfaces.

In this study, the amount of free Cur in the produced HNPs was measured spectrophotometrically, and the EE% of the Cur/CD-HNPs was ascertained following centrifugation [17]. Cur was first complexed with cyclodextrin at a 1:1 molar ratio, a well-established stoichiometry documented in several studies for forming stable inclusion complexes [29, 34]. Cur/CD-NPs showed high encapsulation efficiency, with free drug molecules at < 1% (0.8% ± 0.7%) and encapsulated Cur at 90.2% ± 3.1%. Hence, when 1 mg/mL Cur was used, approximately 0.920 mg/mL was incorporated into the HNPs, with a total biomaterial concentration of 72.5 mg/mL. The Cur loading capacity in the Cur/CD-HNPs was 43.59 ± 1.72%, showing that approximately 1000 mg of NP could encapsulate 435 mg Cur. The CUR incorporation achieved here is relatively high compared with previous investigations on mixed nano systems [54]. Pluronic-86 plays a crucial role in shell-core integration, generating the forces necessary for assembly through the attraction of the alkyl groups of the polymer to the aromatic groups of Cur [55, 56]. This could enhance the Cur-polymer interaction sites and HNPs loading capacity. Chol also plays a strategic role in the composition of HNPs. Surfactant molecules (hydrophobic components of the chain) can be adsorbed or partially intercalated between lipid chains during nanoprecipitation. Hence, Chol enhances drug permeability and reduces HNPs loss [31]. A high EE is ideal for natural medicinal drugs such as Cur, which requires a greater dose for potency and, hence, an extensive number of nanoparticle carriers. Chol optimized the bioavailability of Cur. Thus, the presented technique is beneficial for loading more Cur than other typical NP formulations.

### Developed hybrid nanoparticle physicochemical and structural properties

#### Particle size and Z-potential

**Fig. 2:**
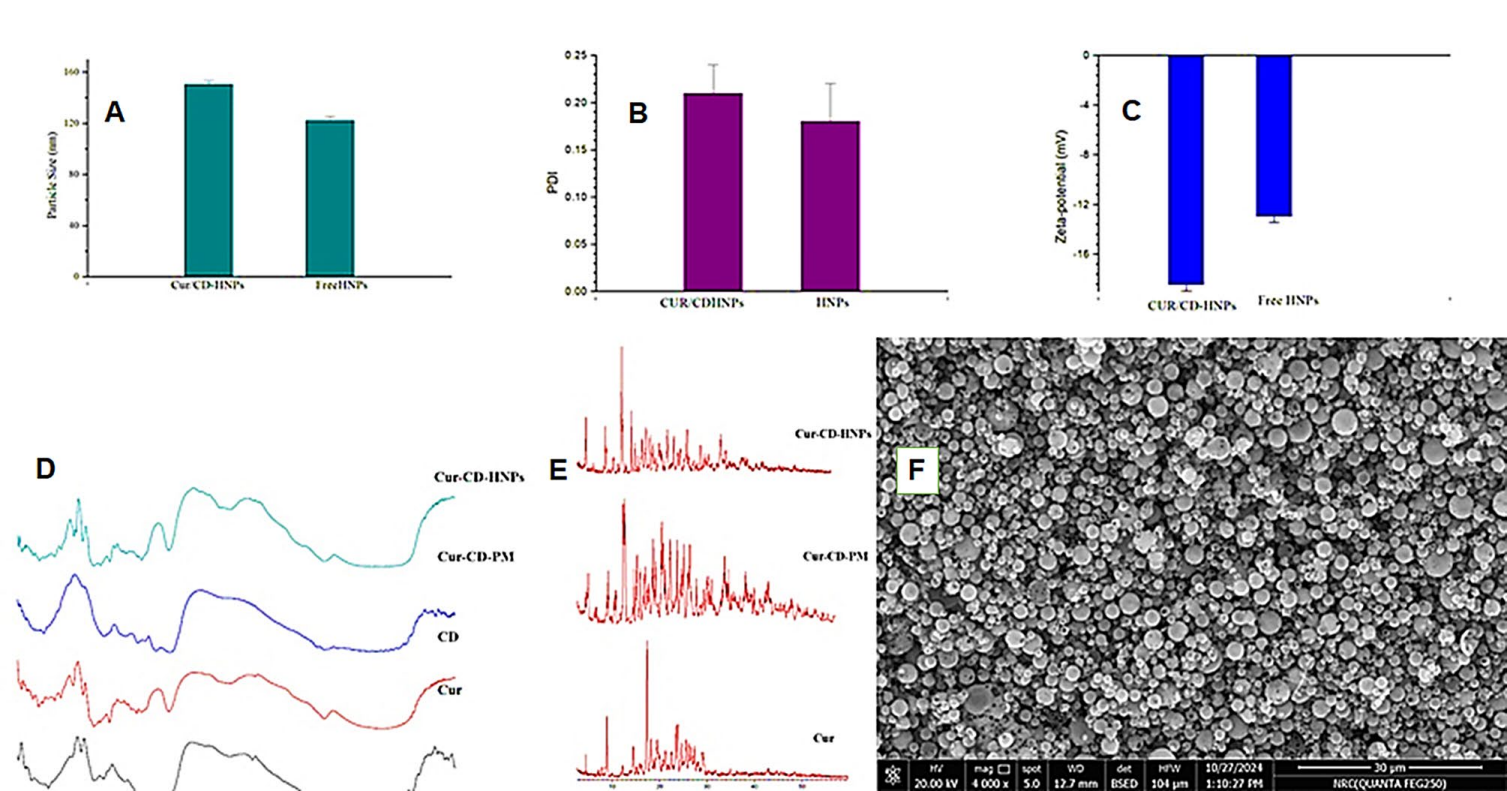
Characterization of Free NPs and Cur/CD-HNPs (**A**) particle size and PDI (**B**) Zeta-potential. Data are means ± SDs (*n* = 3), (**C**) PDI: polydispersity index (**D**) FTIR analysis of Cur, CD, Cur/CD-PM and Cur/CD-HNPs (**E**) XRD pattern for Cur, Cur/CD-PM and Cur/CD-HNPs. (**F**) SEM images of Cur/CD-HNPs

Determining the particle size is crucial for improving the solubility of unstable, poorly soluble medications because it directly affects their stability and solubility [57]. Figures 2A, B, and C illustrate the average particle sizes of 122.2 ± 2.8 nm and 150.5 ± 2.8 nm, polydispersity indices of 0.18 ± 0.02 and 0.20 ± 0.03, and zeta potentials of − 14.9 ± 0.61 mV and − 18.5 ± 0.59 mV for the formulated free HNPs and Cur/CD-HNPs, respectively. The Cur/CD-HNPs showed a marginal increase in size and PDI compared with the free HNPs. Similar to a previous study, curcumin encapsulation led to a slight increase in the particle size [58]. The authors have previously reported curcumin nanoparticles with particle sizes of approximately 114.9 ± 1.36 nm [59], while other studies have documented sizes around 182.27 ± 7.53 nm [60]. The attained monomodal and monodisperse population of HNPs (PDI = 0.2 ± 0.04) can be attributed to the incorporation of Pluronic-F68. Pluronic-F68 could reduce the surface tension of HNPs with a progressive decrease in size (Z-average less than 200 nm) owing to its high HLB value (26) and known affinity for lipidic structures [33]. The nanostructured Cur/CD-HNPs synthesized in this work offer a significant advantage over previous studies that found Cur and β-CD inclusion complexes to be blocky structures with micron sizes [61]. Hence, the narrow particle-size distribution, high zeta potential, and adequate encapsulation efficiency of the Cur/CD-HNPs are advantageous for Cur delivery and use.

#### Fourier-transform infrared spectral analysis

FTIR analysis was carried out to detect the molecular variations in Cur that could result from interactions with the excipients. In this study, the FTIR spectrum of Cur showed specific absorption bands (Fig. 2D). They correspond to an–OH phenolic stretching vibration at ~ 3510.00 and 3330.26 cm^− 1^, a C-H stretching aromatic group at ~ 3006.16 and 2950.72 cm^− 1^, a C-H stretching band of methyl group at ~ 2840.68 cm^− 1^, C = O stretching group in the curcumin’s alkene chain at ~ 1620.77 cm^− 1^, C-C stretching group at ~ 1510.27 cm^− 1^, and CH_2_ scissoring group in the alkene moiety at 1376.62 cm^− 1^. The C-O stretching band of the benzene ring in Cur was also observed at 1282.06 cm^− 1^ and which is characteristic of Cur. Also, aromatic C-H bands at 1204.90 cm^− 1^ and 1143.10 cm^− 1^ in-plane deformation and aromatic C-H bands at 1025.46 cm^− 1^ out-of-plane deformation were observed. The absorption bands also included a CH_2_ alkene band at 963.56 cm^− 1^, a CH bending band at 856.06 cm^− 1^ and 814.32 cm^− 1^, an aromatic C-C-C bending at 575.71 cm^− 1,^ and an aromatic C-C bending out of plane at 468.35 cm^− 1^. From the FTIR analysis results, the spectra of Cur/CD-PM and Cur/CD-HNPs matched that of Cur. Hence, the Cur bands were maintained in Cur, CD, Cur/CD-PM, and Cur/CD-HNPs, indicating no change in their molecular structure [61].

#### Determination of variation in the crystal structure using XRD

X-ray diffraction (XRD), a method for crystal phase determination, was used to obtain data on the physical properties of the samples. XRD analysis was performed on the Cur, Cur/CD-PM, and Cur/CD-HNPs (Fig. 2E). The diffractogram of Cur shows its crystalline nature, indicated by two prominent peaks with intensities at 2θ = 8.90° and 17.34°. In contrast, the diffractograms of Cur/CD-PM and Cur/CD-HNPs showed a significant reduction in these peaks, indicating the existence of Cur in an amorphous form. The relative degree of crystallization (RDC) was calculated using the simple equation RDC = I_sample_/I_drug_ [40], where I_sample_ represents the height of the peak of the investigated sample (PM or HNPs), and I_drug_ represents the height of the peak at the same angle for drug Cur. Hence, the RDC values for the PM and HNPs were 0.058 and 0.79, respectively. These results confirmed the significantly reduced crystallinity of Cur [61]. This transformation indicated distinct molecular aggregation patterns of Cur and CD, which enhanced their potential for antimicrobial and drug delivery applications. Because amorphous drugs generally exhibit greater water solubility and bioavailability than their crystalline counterparts, the reduction in curcumin crystallinity leads to an improved dissolution rate [62].

#### Scanning electron microscopy analysis

The SEM micrograph (Fig. 2F) reveals the detailed surface morphology of the Cur/CD-HNPs. The particles displayed a highly uniform and predominantly spherical shape, with a dense and discrete distribution (Fig. 2F). The uniformity and smooth surface texture observed are favorable characteristics for topical applications, as they contribute to improved dispersibility, controlled release, and enhanced skin permeation. The absence of visible aggregation or deformation further suggests successful spray-drying conditions and good physical stability of the hybrid nanoparticles.

### Stability evaluation of the nano-encapsulated cur

#### pH stability assessment

**Fig. 3:**
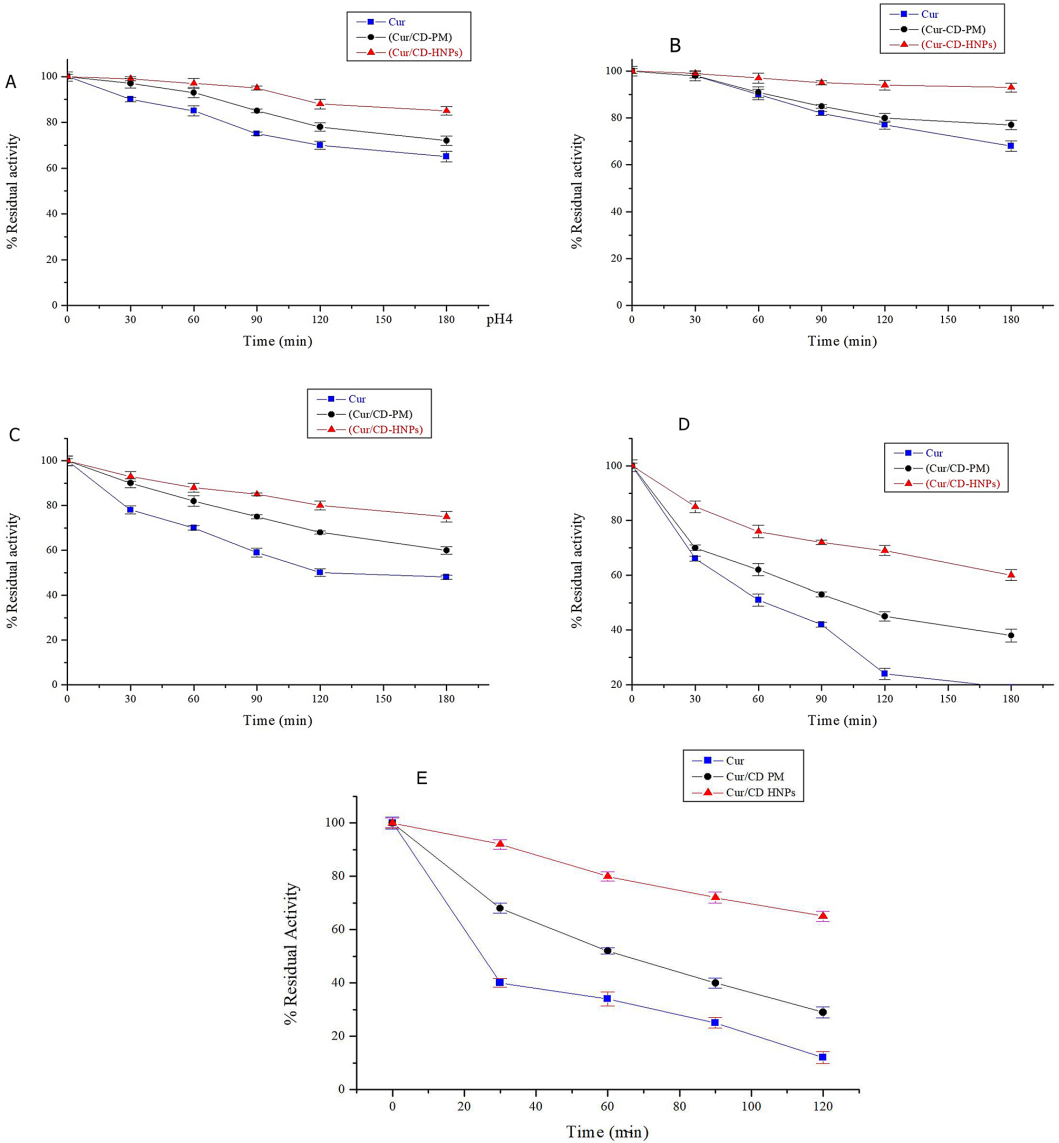
Physical stability characterization. (**A**-**D**) pH stability of Cur and Cur/CD-HNPs. **B**–**E** represent different pH conditions of 4.0, 6.0, 8.0, and 10.0, respectively. (**E**) Thermal stability of Cur and Cur/CD-HNPs incubated at 100 ^o^C for 120 min. Values are expressed as means ± SDs (*n* = 3)

Figure 3 (A-D) shows the retention rate of Cur at various pH values (pH 4–10). All samples showed a decreasing tendency in the Cur content over time, with different pH values. Furthermore, the rate of Cur deterioration in the samples increased progressively as the acidity or alkalinity increased. However, even under unfavorable conditions, Cur/CD-HNPs maintained a higher Cur content than free Cur or Cur/CD-PM. Notably, the rate of Cur loss in acidic environments was comparatively lower than that in alkaline environments, whereas Cur/CD-HNPs retained over 55% of the Cur after acidic incubation. Our results showed that Cur/CD-HNPs have a high degree of stability across a variety of acid-base environments, suggesting that they could be useful for delivering bioactive substances to various biological sites. Previous studies have also demonstrated that encapsulating Cur in nanoparticles can improve the pH stability [43]. In an alkaline aqueous solution (pH ≥ 7.0), Cur is extremely susceptible to chemical degradation [63]. This effect is explained by the partial deprotonation of the hydroxyl group of Cur in neutral to alkaline environments, which increases the water solubility and chemical reactivity of the compound [64].

#### Thermal stability study

Figure 3 (E) presents the Cur retention rates of the tested samples, given as RR%, after 120 min of continuous heating at 100 °C. The results depicted in Fig. 3 indicate that within the first half hour, Cur in the Cur/CD-PM and Cur/CD-HNP samples exhibited a swift declining trend, with the breakdown of free Cur surpassing 50%. In contrast, the RR% of the Cur/CD-PM and Cur/CD-HNPs were 65.92 ± 2.16% and 83.48 ± 2.35%, respectively. Cur was retained following the heating treatment in the following order: Cur/CD-HNPs > CUR/CD-PM > Free CUR. The ability of the Cur/CD-HNPs to inhibit the thermal denaturation of Cur was statistically significant (*p* < 0.05) compared to that of the Cur-CD-PM. Higher temperatures decrease the CUR stability, which may be crucial for the processing, storage, and use of CUR-containing products. Notably, compared to free Cur, Cur/CD-PM showed a greater RR, perhaps because of the presence of CD, which greatly improved the thermal stability of Cur [43]. These findings also imply that Cur is effectively protected by nanoencapsulation, perhaps because of the more constrained nanoenvironment offered by HNPs [29]. Thus, the deterioration of the Cur was reduced, and the thermal stability increased at high temperatures. The enhanced heat stability of the cur-encapsulated nanoparticles has been reported previously [65].

#### Drug retention and long-term stability study

Freeze-drying is regarded as a feasible test to meet the long-term stability requirements. Following the ICH recommendations (http://www.ich.org), investigations were performed to assess the long-term stability of the developed HNPs. Freeze-dried Cur/CD-HNPs appeared stable after three months of storage at 25 °C and 60% RH, as the dried cakes exhibited no collapse or shrinking. Moreover, assessments of the particle size and polydispersity index showed that there had been no significant changes (*P* < 0.05) (Table 1). Cur/CD-HNPs were freeze-dried without adding an excipient because CD functions as a cryoprotectant and facilitates reconstitution [66]. The data presented in Table 1 shows that after 90 days of storage, the EE% was 96.2% ± 5.6%. This finding unequivocally demonstrates that the Cur/CD-HNP formulation protected the Cur against deterioration and/or leakage during storage. By locating the Cur in the β-CD cavities, hybrid nanoparticles were created, which made the Cur more stable for storage. This could be because of the high concentration of β-CD utilized in the nanoencapsulation process, which successfully sealed off the air and stopped the oxygen oxidation-induced Cur degradation. The surfactant was organized into micelles during Chol nanoprecipitation in an aqueous solution of Pluronic-F68 at 45 °C. This makes Chol-based HNPs more flexible. The HNPS exhibited a more cohesive reorganization of the components, ensuring stability at dilution. Similar results were observed in a previous study, where the creation of nanoparticles with β-CD increased the storage stability of catechins [28]. Therefore, it is reasonable to conclude that CD-based NPs have great potential for improving Cur delivery.

**Table 1:** Storage stability data of formulated Cur/CD/-HNPs. Data means ± sds (*n* = 3), PDI: polydispersity index, EE encapsulation efficiency

Timepoint (days)	Particle size (nm)	PDI	EE%
0	150.5 ± 2.25	0.21 ± 0.03	100% ± 5.6%
15	150.5 ± 2.52	0.21 ± 0.04	99.8% ± 4.9%
30	151 ± 3.65	0.22 ± 0.02	99.2% ± 4.9%
60	152.5 ± 2.35	0.22 ± 0.05	98.3% ± 4.4%
90	155 ± 3.12	0.23 ± 0.03	96.2% ± 5.6%

### Anti-inflammatory properties

**Fig. 4:**
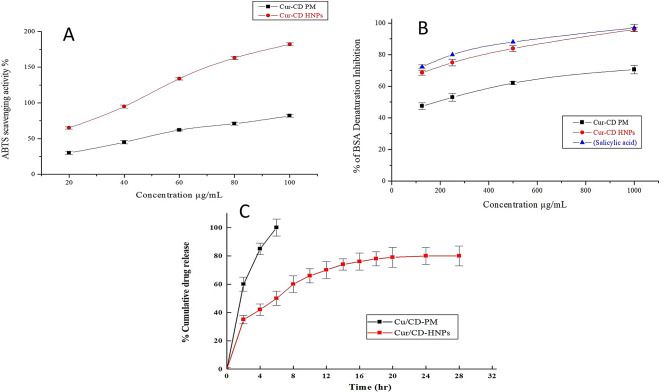
Anti-inflammatory and radical scavenging activity evaluation. (**A**) Comparison of the concentration-dependent anti-inflammatory activity assay of Cur/CD-PM and Cur/CD-HNPs. (**B**) Comparison of the concentration-dependent in vitro radical scavenging assay (ABTS assay) of Cur/CD-PM and Cur/CD-HNPs. (**C**) The cumulative percentage release of curcumin from Cur/CD-HNPs as compared to Cur/CD-PM in PBS (0.01 M). An Initial burst effect followed by a sustained release effect up to 24 h. Values are expressed as Mean ± SD (*n* = 3)

The anti-inflammatory properties of Cur/CD-PM and Cur/CD-HNPs were investigated using the BSA denaturation assay. Figure 4-A shows the anti-inflammatory activities of 125, 250, 500, and 1000 µg/mL Cur. The data showed a progressively increasing inhibition rate with increasing Cur concentrations. The anti-inflammatory activities of Cur/CD-PM and Cur/CD-HNP samples ranged from 47.45 ± 2.1% to 70.55 ± 2.7% and 68.59 ± 1.5% to 97.93 ± 1.24%, respectively. Compared to Cur/CD-PM, Cur/CD-HNPs exhibited greater anti-inflammatory effects. Additionally, at high concentrations of Cur, the protective effect against albumin denaturation was statistically significant (*p* < 0.05) compared with that of the standard drug. The results also showed that the IC50 value of the Cur/CD-HNPs was highly significant (*P* < 0.05) (Table 2). NSAIDs, such as salicylic acid, can help reduce inflammation by preventing BSA from breaking down at pH levels that are too high or too low. Protein denaturation in pathological tissues results in the production of autoantigens, which in turn cause various inflammatory reactions. Electrostatic variations in the hydrogen, hydrophobic, and disulfide bonds are part of the denaturation mechanism. Usually triggered by a wound, the inflammatory response helps the body to remove debris from the injured area, shields it from incoming microbes, and signals the movement of cells required for repair processes. However, prolonged inflammatory responses destroy repaired tissues and leave scars [67]. Therefore, it is possible to reduce the inflammatory activity by inhibiting protein denaturation. Hence, the results presented here show that Cur/CD-HNPs can reduce inflammation and accelerate wound healing.

**Table 2:** IC_50_ values for the anti-inflammatory activity assay of Cur/CD-PM and Cur/CD-HNPs

Tested samples	IC_50_ of BSA denaturation assay (µg/mL)
Cur/CD-PM	148.59 ± 1.16*
Cur/CD-HNPs	104.73 ± 1.12*
Salicylic Acid	91.23 ± 0.93

Values are mean ± SD (*n* = 3); Level of Significant *p* < 0.05 are considered as significant (*)

### Antioxidants and the radical scavenging efficiency

To evaluate the effect of encapsulation on the antioxidant capacity of Cur, the ABTS scavenging method was used to measure free Cur, CUR/CD-PM, and Cur/CD-HNPs. The ABTS assay is a simple, quick, accurate, and repeatable process. Electron transfer is involved in the reaction of ABTS^•+^ radicals [7]. The data shown in Fig. 4B demonstrate that Cur is an efficient ABTS ^+^ radical scavenger in a concentration-dependent manner (20–100 µg/mL), and its scavenging capability gradually increases with increasing concentrations. The ABTS^+^ scavenging potential varied significantly between the Cur/CD-HNPs and Cur/CD-PM. The ABTS radical scavenging rate of Cur/CD-HNPs was 100% at 40 µg/mL. In addition, the IC_50_ values for Cur/CD-PM and Cur/CD-HNPs were 21.27 and 12.85 µg/mL, respectively. Cur/CD-HNPs demonstrated a lower IC_50_ and a significantly stronger antioxidant activity than Cur/CD-PM. The results of the One-way ANOVA demonstrated a substantial (*p* < 0.05) difference in the IC_50_ of Cur nanoparticles compared to Cur/CD-PM, indicating considerable enhancement of antioxidant activity through nanoencapsulation in HNPs. Cur/CD-HNPs enhanced antioxidant activity owing to their increased surface area, improved water solubility, and exposure to more hydroxyl groups from Cur molecules, thereby reducing free radicals. These findings align with earlier research reports on nanoparticle formulations [43, 68, 69], where comparable findings were revealed in their studies after Cur encapsulation.

### In vitro antibacterial properties

Pathogen-induced wound infections are a significant determinant of wound healing [70, 71]. Using the disc diffusion method, we assessed the antibacterial efficacy of Cur/CD-PM and Cur/CD-HNPs against four typical pathogenic bacterial strains in vitro. Both Cur formulations inhibited all the tested microorganisms. Compared with Cur/CD-PM, Cur/CD-HNPs mediated a greater inhibitory zone on S. aureus and B. *subtilis* viability, as shown in Table 3, and exhibited significantly higher antibacterial activity (*p* < 0.05). This could be partly explained by the small size of the HNPs and high surface-to-volume ratio, which allows for better transit through biological barriers and favorable interactions with pathogens. The Plain hydrogel did not exhibit an inhibitory zone, indicating that the hydrogel base lacked antibacterial activity against the tested microorganisms. Our results indicate that Cur/CD-HNPs are more effective against gram-positive strains than against gram-negative pathogens (*E. coli and P. aeruginosa*). Similar outcomes were reported by [46, 70], who examined the antibacterial activity of bulk Cur and nano curcumin against Gram-positive and Gram-negative bacteria, respectively. This is presumably related to how nanoparticles interact with microbial DNA and the lipid layers of cell membranes to inhibit bacterial growth [72]. However, the antibacterial effectiveness of nanoparticles against gram-negative strains is diminished by their repulsion by negatively charged moieties on the bacterial surface during contact. Consequently, Gram-positive bacteria are more vulnerable to antimicrobial treatment than Gram-negative bacteria. The biological traits of bacteria, their capacity to produce exopolysaccharides, and variations in the permeability of the outer membrane when paired with these factors may account for the diversity in the antibacterial action of Cur in HNPs against various bacterial strains [73]. Similarly, Rai et al. and Sankhwar et al. [74, 75] reported enhanced in vitro antibacterial efficacy of nano-curcumin against gram-positive bacteria compared with that against gram-negative bacteria. Furthermore, the antibacterial activity of Cur/CD-PM and Cur/CD-HNPs did not change significantly (*p* ≤ 0.05) from their initial values after a 21-day storage period; however, Cur/CD-HNPs maintained their antibacterial activity.

**Table 3:** Diameters of Inhibition zones for Cur/CD-PM and Cur/CD-HNPs against microbial pathogens using the disc diffusion method (in vitro) with Day(s) intervals (mean ± SD, *n* = 3)

		Inhibition zone (mm)			
		Cur/CD- PM		Cur/CD-HNPs	
	Bacteria strain	day 0	day 21	day 0	day 21
Gram-positive	*S. aureus*	22.25* ± 1.26	24.83 ± 1.14	25.80* ± 0.84	25.60 ± 1.14
	B. *subtilis*	21.53*±0.91	20.47 ± 0.98	23.98*±0.97	22.45 ± 1.1
Gram-negative	*E. coli*	18.04* ± 1.14	17.23 ± 0.50	20.33* ± 0.58	19.40 ± 1.99
	P.*aeruginosa*	19.32*±	18.24 ± 0.78	21.23*± 0.85	20.89 ± 0.96

(*) indicates statistical significance at (*P* < 0.05)

### In vitro drug release studies

As shown in Fig. 4C, the Cur/CD-PM, used as a control, demonstrated a rapid release profile, with 65.58% of the Cur released within the first 30 min and achieved complete release by 2 h. This indicated poor stability and unfavorable bioavailability. Similar results for Cur were reported before [76]. The in vitro release study of Cur/CD-HNPs demonstrated a biphasic release pattern characteristic of nanoparticulate drug delivery systems. An initial burst release of approximately 35.45% of the encapsulated Cur was observed within the first 2 h. This was followed by a sustained release phase, with a cumulative release reaching 82.35% over 24 h. The accelerated release at first may result from the diffusion of surface-associated drug molecules and possibly the increased hydrophilicity following hydration, causing structural loosening due to hydration-induced expansion. Consequently, CUR encapsulated within the HNPs showed a markedly slower release rate, indicating enhanced stability upon encapsulation within the HNPs. This improved stability is likely due to the hydrophobic interactions between HNPs and CUR, which shield the drug from degradation [60]. The sustained release observed may be attributed to the diffusion of drug molecules through the lipid matrix of the HNPs into the dissolution medium. Additionally, pluronic triblock copolymers. Hydrophobic blocks reinforce the molecular network and stabilize the NPs through intermolecular interactions [77]. Comparable results characterized by an initial burst followed by a prolonged release from polymeric nanocarriers have been reported in previous studies [78], supporting our findings. For instance, Ahmad et al. reported a similar biphasic release from a curcumin nanoemulsion, with an initial burst followed by sustained release [38]. Additionally, Hong et al. [29] observed a pH-dependent release from curcumin-loaded hybrid nanoparticles, with a cumulative release of 52.78% at pH 7.4 over 7 days, further supporting the diffusion-controlled release mechanism. Incorporating Tween 80 in the release medium was crucial for maintaining the sink conditions and enhancing the solubility of Cur, a hydrophobic compound. This approach is consistent with methodologies employed in similar studies to ensure accurate assessment of release profiles [48].

The initial rapid drug release may play a key role in establishing the high concentration gradient necessary for efficient transdermal drug delivery. Additionally, the sustained release observed in this study suggests that Cur/CD-HNPs can provide prolonged therapeutic effects. Such a release profile is advantageous for maintaining therapeutic drug levels over extended periods, potentially reducing dosing frequency and enhancing patient compliance, making them suitable candidates for applications requiring extended drug release, such as in chronic wound management.​.

### Cytotoxicity/cell viability evaluation

**Fig. 5:**
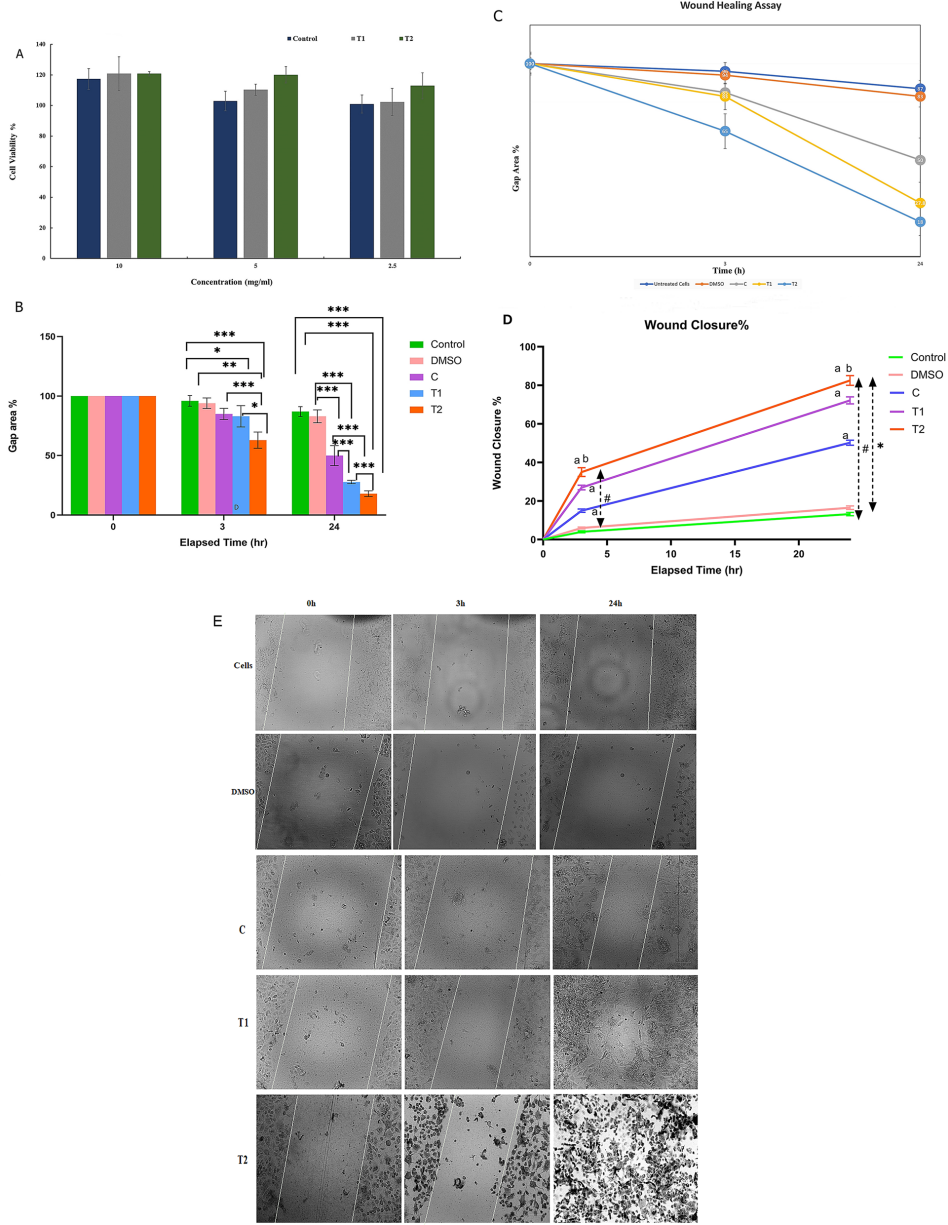
Cytotoxicity and wound healing studies *In vitro.* (**A**) Effect of Curcumin-loaded HNPs on the viability of fibroblast cells (HSF) evaluated by MTT assay (**B**) Quantification of wound area, (**C**) Cell migration speed (**D**) Graphical illustration of wound closure% versus elapsed time in hours for all treatments. Data are represented as mean ± SD. ^a, b^ denote significance against control and C groups, respectively at *p* < 0.05. Whereas for elapsed time, ^#, *^ denote significance across time zero and 3 h, respectively. (**E**) Optical micrographs of scratch test in vitro using HSF cells at different time points photographed with light inverted microscopy. *Indicates significance at *p* < 0.05. Values are expressed as Mean ± SD (*n* = 3). (****indicates significance at *p* < 0.0001, *** indicates significance at *p* < 0.001 and * indicates significance at *p* < 0.05)

Biocompatibility is the primary consideration for wound dressings. When in direct contact with wounds, ideal wound treatments would not cause an immune response but could promote wound healing by enhancing cell vitality and proliferation [79]. This study tested the biocompatibility of HNP according to ISO 10,993–5 “Biological evaluation of medical devices- Part 5: Tests for in vitro cytotoxicity”, which requires at least 70% cell viability for the indicated usage. The cytotoxicity of the Cur/CD-PM hydrogel matrix (T1) and the Cur/CD-HNPs hydrogel matrix (T2) was evaluated using HSF cells for potential cytotoxicity at concentrations of 10, 5, and 2.5 mg/mL (Fig. 5A). The results revealed that the cell viability in all groups surpassed 70% and exhibited excellent biocompatibility at the tested concentrations, indicating their safety for use in our target application. Despite its broad-spectrum medicinal uses, pure Cur has demonstrated substantial cytotoxicity at high concentrations. Cur induces cell death in pure and non-carrier-based delivery systems [80]. However, in our study, the bio-safe nano-precipitation methodology utilized in Cur NPs preparation was demonstrated to mitigate this cytotoxicity compared to pure Cur. This risk was greatly mitigated when Cur was incorporated into the Cur/CD-HNPs, where it did not impede fibroblast growth and cell proliferation owing to biocompatibility. This high biosafety may also be attributed to the green synthesis method, which uses FDA-approved materials and does not introduce any toxic substances. Furthermore, the inclusion of the Cur/CD-HNPs in the hydrogel matrix enhanced its stability and helped regulate its disintegration rate. Madamsetty et al. demonstrated that dressings based on chitin can enhance tissue regeneration by aiding in wound contraction and controlling the release of inflammatory mediators [81]. Moreover, chitosan-gelatin hydrogels encourage the in vivo development of tissues, including the skin, cartilage, and bone [43].

### Wound healing scratch assay

A wound healing scratch assay was performed to investigate the potential stimulatory effect of Cur/CD-HNPs (T2) on cell migration in vitro. This procedure enables the examination of HSF cell migration in a two-dimensional confluent monolayer under in vitro test conditions [82]. As shown in Fig. 5B and C, the T2 group achieved faster scratch closure than the control and T1 groups. Within 3 h, the scratch area treated with T2 showed a significantly greater number of migrating fibroblasts than that of the control or T1 (*p* ≤ 0.05). Two-way ANOVA followed by Tukey’s post-hoc test was used for the wound closure data. Compared with the starting time of the experiment, a significant difference was reported across all treatment groups after 3 and 24 h (denoted as #) at *p* < 0.05. Similarly, there was a significant increase in wound closure after 24 h compared to the 3 h elapsed time (denoted as *) at *p* < 0.05. Across the treated groups, treatment with either T1 or T2 hydrogels significantly improved wound closure compared with the control group (denoted as a). The T2 hydrogel demonstrated a significantly greater healing effect than T1, compared to the blank control group C (denoted as b), with statistical significance at *p* < 0.05 (Fig. 5D). Quantification analysis revealed that after 24 h of incubation, the percentages of wound area gaps in the blank, T1, and T2 hydrogels were 50%, 27.8%, and 18%, respectively (Fig. 5C). The migration rate of the T2 hydrogel was significantly higher than those of the blank control and T1 hydrogels (*p* < 0.05) (Fig. 5C and E). HNPs’ high surface-area-to-mass ratio of HNPs facilitates effective cell-Cur interactions on a nano-dimensional scale. Collectively, Cur/CD-HNPs substantially enhanced fibroblast migration, thereby facilitating scratch-gap closure and wound healing (Fig. 5E).

The scratch assay is a widely employed method for investigating the impact of various medications and biological factors on keratinocytes and fibroblasts. In fibroblasts, scratching increases reactive oxygen species, Nrf2 protein, and stress response genes such as heat shock protein 70 and heme oxygenase-1 [80, 83]. Cur stimulates cell motility, supports skin tissue regeneration, and wound healing [84]. Additionally, mechanical damage resulting from scratching triggers the release of chemicals from the surrounding cells. However, scratching leads to elevated intracellular calcium levels in injured cells. The propagation of calcium waves extends to cells distant from the wound site and is transmitted through cell-cell junctions [85]. Increased intracellular calcium levels promote signaling pathways and alter gene expression [86]. Previous research studies have shown that Cur can lower intracellular calcium levels in colorectal cancer cells in a dosage-dependent manner [87]. Hence, it accelerates the wound-healing process. In a Previous study in murine cell lines, curcumin nanoparticles showed an 85.62% increase in wound healing capacity, demonstrating their efficacy in encouraging tissue repair [88]Another study has highlighted that in human dermal fibroblasts, curcumin nanoparticles were effective nanocarriers for enhancing wound healing processes [89].

### In vivo wound healing study on the burn model in rats

**Fig. 6:**
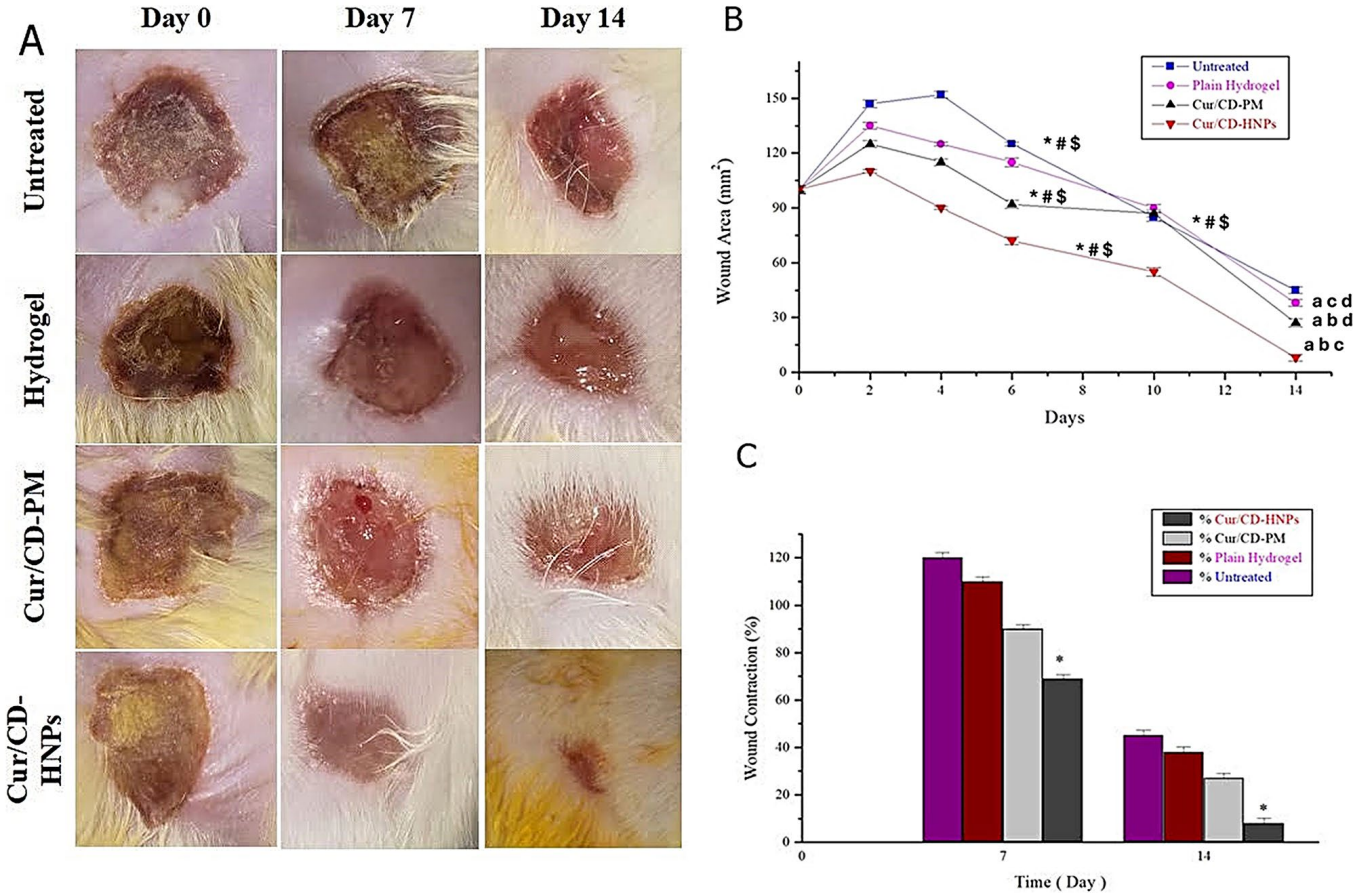
In vivo evaluation of Cur/CD-HNPs accelerated burn wounds healing model in vivo at different periods (days): (**A**) Representative images of burn wounds at different days (0,7 and 14 days) in comparison with untreated/normal saline, plain hydrogel, Cur/CD-PM and curcumin-loaded HNPs (Cur/Cd-HNPs); (**B**) Graphical illustration of Wound area across the experiment time (14 days) for all treated groups. Data are represented as mean ± SD. ^a, b,c, d^ denote significance against control, plain, CUR-CD/PM and CUR-CD/HNPs respectively at *p* < 0.05. Throughout the experiment period, ^*,#,$^ denote significant differences of the treated groups over day 6, going through day 10 and 14 respectively at *p* < 0.05. (**C**) Day 7 and 14 post-treatments, (measured as percent of initial area (*n* = 8)) Wound size data are presented as mean ± SD. Statistical significance compared with the untreated group was done. *Indicates significance at *p* < 0.05

The wound healing efficacy of Cur/CD-HNPs was systematically evaluated using a burn wound model in Wistar rats. The animals were divided into four groups: the first one was the untreated group, group two was subjected to the plain hydrogel, groups three and four were assigned to the Cur/CD-PM hydrogel and the Cur/CD-HNPs hydrogel, respectively. The wound closure percentage and epithelialization time were used as primary indicators of healing progression (Fig. 6A). Notably, wounds treated with the Cur/CD-PM and Cur/CD-HNPs hydrogels exhibited significantly reduced inflammatory expansion zones (approximately 125.1 ± 3.14% and 110.0 ± 2.6%, respectively) compared to the untreated (147.9 ± 4.3%) and plain hydrogel groups (134.7 ± 4.41%). Among the treated groups, the Cur/CD-HNPs hydrogel demonstrated the most remarkable enhancement in wound healing, resulting in the maximum reduction in wound area (*p* < 0.05) (Fig. 6B). Statistical analysis using two-way ANOVA followed by Tukey’s post hoc test confirmed that starting from day 4, all treated groups exhibited a significant improvement in wound closure, with the most pronounced effects observed at later stages, particularly on day 14. Compared to the control group, wounds treated with Cur-loaded HNPs exhibited significantly faster healing rates and smaller wound areas (Fig. 6B). By day 7, the Cur/CD-HNPs hydrogel had already induced a statistically significant acceleration in wound closure (Fig. 6C). By day 14, nearly complete wound closure was observed in the Cur/CD-HNPs hydrogel-treated group, whereas wounds treated with Cur/CD-PM hydrogel achieved only 77% closure, indicating a delay in the healing process (Fig. 6B). Similarly, prior research has shown that antioxidant medications increase the wound healing impact [2, 35, 42].

The superior wound healing potential of the Cur/CD-HNPs hydrogel could be attributed to the physiological lipid composition of the HNPs, which interacted with the stratum corneum to reorganize its lipid structure, thereby enhancing skin barrier repair and accelerating tissue regeneration. This process facilitates drug molecule penetration into deeper layers, promoting wound contraction and re-epithelialization [35]. Drug absorption via the skin is also facilitated by the small size of the NPs, which increases their adhesiveness and surface contact area. Wound dressings are usually made with hydrogels to provide warm, moist wound healing environments that boost healing rates [5, 84]. Hydrogels hydrate wounds, aid autolytic debridement, and enhance cellular motility. Similar to the extracellular matrix, hydrogels function as barriers to reduce microbial invasion, enhance oxygen permeability at the wound site, and thereby accelerate healing [43]. In addition, these dressings allow autolytic debridement without harming the epithelial cells. Previous reports have described the wound healing effect of Cur-loaded NP [90], which demonstrated a notably faster rate of wound closure. Collectively, these findings underscore the significant therapeutic potential of Cur/CD-HNPs in facilitating rapid and efficient wound healing.

### Histopathological analysis and tissue regeneration

**Fig. 7:**
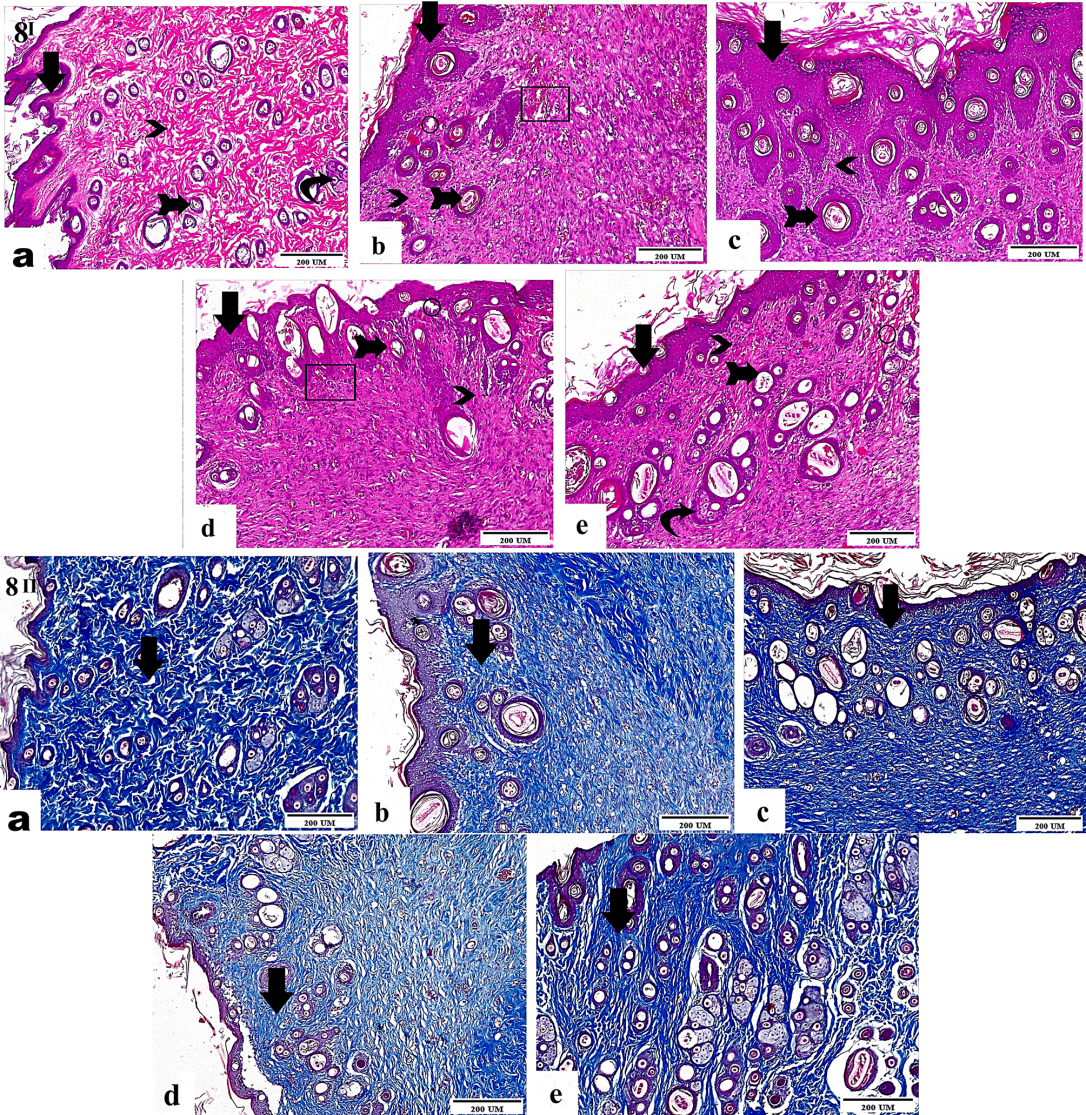
(I) Histopathological examination of H&E stained images for different groups illustrating **a**) Normal healthy skin with intact connective tissues (arrows), hair follicles, and skin glands. **b**) Untreated skin revealing interstitial hemorrhage (rectangle) and edema (circle). **c**) Positive control skin showing thickened migrating epidermal cells (arrows). **d**) Cur/CD-PM treated skin showing improved newly formed epidermal cells, with fewer hemorrhage and lesser edema. **e**) Cur-CD HNPs treated skin showing higher numbers of migratory epidermal cells and restored structure of connective tissues and skin glands. Magnification: 100x. (II) Comprehensive microscopical images of skin tissues of all groups stained with Masson’s Trichome stain showing **a**) Normal intact healthy skin, **b**) Untreated skin showing less collagen stain, **c**)Positive control skin demonstrating high collagen staining. **d**)Cur/CD PM treated skin manifesting improved collagen staining and organized connective tissue. **e**)Cur/CD HNPs treated skin spotting remarkably high collagen staining and normal fibrous connective tissues. Magnification:100x

Histological analysis further supported the wound-healing potential of the Cur/CD-PM (T1) and Cur/CD-HNPs (T2) loaded into the hydrogel matrix. Histological examinations were performed on day 14 after treatment when robust skin closure was noted. Figure 7I. Photomicrographs of the H&E-stained images of different testing groups. The healthy control skin group had an intact epidermal layer, a typical dermis layer, normal hair follicles, and integral skin glands with no detectable inflammation. The negative control group exhibited inflammatory edema, increased dermal thickness, and numerous inflammatory cells invading the adnexa and epidermis. Similarly, the positive control and T1 groups had a high level of inflammatory cell infiltration in the dermis (Fig. 7I). Compared to the other groups, the improvement in the histopathological characteristics of the T2-treated animals was more significant. T2-treated animals showed more re-epithelialization and more intense epithelial lines, indicating more mature epidermal tissue. Additionally, the granulation tissue exhibited greater size and thickness. Thus, the T2-treated groups exhibited enhanced granulation tissue maturation compared with the other groups. More significantly, T2 treatment increased the regeneration of the skin glands and fibrous connective tissue in wound tissues (Fig. 7I). Wound healing is a complex and dynamic process that involves phases of inflammation, proliferation, and maturity, with the involvement of various cell types, such as keratinocytes, endothelial cells, immune cells, and fibroblasts, which play overlapping roles. Prolonged tissue inflammation creates excessive levels of cytotoxic enzymes, inflammatory mediators, free radicals, and cytokines, which harm the surrounding tissue cells. The overproduction of free radicals also results in oxidative stress and cytotoxic consequences, which slow wound healing. Thus, the use of anti-inflammatory and antioxidative drugs to reduce chronic inflammation and free radicals may improve cell proliferation and wound healing [91, 92]. The development of granulation tissue and its transformation into a primary fibrous scar are crucial physical features of wound healing [93]. Cur functions principally in the proliferative phase of wound healing by enhancing collagen deposition, myofibroblast contraction, re-epithelialization, and fibronectin synthesis [94]. The effects of TGF-β1, inducible nitric oxide synthase, and antioxidant and anti-inflammatory properties may also explain these findings [91]. Wound healing requires neovascularization to carry nutrients and maintain oxygen balance for cell proliferation and tissue regeneration. Cur increases wound-healing neovascularization, possibly due to nitric oxide synthase activity [94]. These findings highlight the capacity of the HNPs formulated to support a controlled and balanced inflammatory phase during wound healing. Additionally, they suggested its potential to enhance tissue revascularization and promote angiogenesis.

Masson’s Trichrome staining (Fig. 7II), and collagen fibers are shown in blue. Following a 14-day course of the treatment, evaluation of the collagen content in the control and Cur-treated wound tissues demonstrated that T2 promoted the production and deposition of collagen. The T2 and T1 treated groups showed increased collagen deposition compared with the control groups. In the T2-treated wounds, the collagen fibers appeared to mature faster and more completely (Fig. 7II). Enhanced collagen synthesis reflects the formulation’s ability to support tissue regeneration, which is a key factor in restoring integrity and function at the wound site. This can also serve as an indicator of its potential efficacy in vivo [95]. This conclusion is supported by the significant increase in the tensile strength and shrinking of wounded tissues. The wound-healing process activates local fibroblasts, resulting in collagen augmentation. Collagen exhibits strong platelet-aggregating activity and is essential for the production of homeostatic plugs. Early increases in collagen synthesis are linked to the early appearance of collagen and may sustain and structure wounds as they heal [96]. These findings indicate the superior potential of Cur/CD-HNPs in accelerating wound healing. Our current histopathological results agree with those of previous studies. Chitosan tamarind-based nanoparticles were reported to improve skin regeneration, increase the production of collagen type I and hyaluronic acid, and decrease tumor necrosis factor levels, all of which have been shown to improve wound healing significantly [12]. In another study, curcumin nanomicelles displayed greater re-epithelialization, increased angiogenesis, and structured granulation tissue in burn sites [97].

## Conclusion

This study highlights the successful development of a novel curcumin-loaded β-cyclodextrin hybrid nanoparticle system (Cur/CD-HNPs) synthesized via nanoprecipitation, offering a highly stable and bioavailable platform for therapeutic delivery. The optimized nanoparticles exhibited a favorable physicochemical profile, including nanoscale size, controlled surface charge, and high encapsulation efficiency, ensuring efficient topical delivery of Cur at therapeutic levels. Through a synergistic host–guest complexation mechanism involving β-CD and Cur within the hybrid matrix, the formulation markedly enhanced curcumin’s solubility, stability, and resistance to degradation, thereby preserving its pharmacological activity. The Cur/CD-HNPs demonstrated potent multifunctional efficacy, exhibiting robust anti-inflammatory, antioxidant, and broad-spectrum antibacterial activities—critical attributes for accelerating wound healing and overcoming key biological barriers. This innovative nano formulation represents a promising strategy for advanced wound care and targeted dermal therapies. The In vitro assays demonstrated biocompatibility and a significant enhancement in fibroblast proliferation and migration, indicative of accelerated tissue regeneration. Complementary in vivo evaluations in burn wound models showed markedly improved wound closure outcomes (*P* < 0.05), driven by rapid epithelialization, enhanced collagen remodeling, and robust neovascularization. These findings underscore the therapeutic potential of the hydrogel-integrated Cur/CD-HNPs as a multifunctional platform for promoting efficient and safe wound healing. Collectively, these findings position Cur/CD-HNPs as a potent wound care nanoplatform that effectively addresses curcumin’s limitations while enhancing its therapeutic efficacy. The optimized formulation shows strong potential for clinical translation in advanced wound healing and regenerative medicine.

### Prospective outlook

While significant progress has been made in leveraging nano-based delivery systems to enhance Cur’s wound-healing efficacy, critical gaps remain in the current research landscape. Despite extensive studies highlighting the pharmacological benefits of Cur-loaded nanocarriers, the absence of comparative analyses across advanced delivery platforms hinders the identification of an optimal formulation for clinical application. Moreover, a lack of large-scale, evidence-based randomized studies limits our understanding of the long-term safety, toxicity profiles, and therapeutic relevance of these innovative systems in both acute and chronic wound healing.

Beyond wound care, the versatility of HNPs holds immense promise for broader medical applications, particularly in combating life-threatening diseases and improving patient outcomes. Future research should explore their potential in regenerative medicine, targeted drug delivery, and systemic disease management. Additionally, a major challenge that remains unaddressed is the industrial production and scalability of nano-formulated Cur for clinical use. Overcoming these translational hurdles will be crucial in advancing Cur nano-delivery systems from laboratory research to real-world therapeutic solutions, unlocking their full potential in next-generation biomedical applications.

## Electronic supplementary material

Below is the link to the electronic supplementary material.

Supplementary Material 1

## Data Availability

Data is provided within the manuscript or supplementary information files.
