# Supplementary material for: Newly designed curcumin-loaded hybrid nanoparticles: a multifunctional strategy for combating oxidative stress, inflammation, and infections to accelerate wound healing and tissue regeneration
Source: BMC Biotechnol. 2025 Jun 19;25:49. doi: 10.1186/s12896-025-00989-z (PMC12180217; doi:10.1186/s12896-025-00989-z)
Supplement: Supplementary file 1 — Supplementary Material 1 [file 12896_2025_989_MOESM1_ESM.docx]

**Newly Designed Curcumin-Loaded Hybrid Nanoparticles: A Multifunctional Strategy for Combating Oxidative Stress, Inflammation, and Infections to Accelerate Wound Healing and Tissue Regeneration**

**Heidi M. Abdel-Mageed ^1*^, Nermeen Z. AbuelEzz^2^, Ahmed A. Ali^1^, Amira E. Abdelaziz^3^, Dina Nada^4^, Sahar M. Abdelraouf ^5^, Shahinaze A. Fouad^6^, Abeer Bishr^7^, Rasha A. Radwan^8^**

**Table S1**: Schematic representation of the samples formulated using nano-precipitation methodology and physicochemical characterization of prepared formulations.

| **Formulation Code** | **Cholesterol (mM)** | **Pluronic F68 (% w/v)** | **Particle Size (nm ± SD)** | **PDI ± SD** | **Zeta Potential (mV ± SD)** | **EE% ± SD** | **Remarks** |
| --- | --- | --- | --- | --- | --- | --- | --- |
| F1 | 0.2 | 0.25 | 245.6 ± 4.3 | 0.34 ± 0.02 | –10.1 ± 0.8 | 61.2 ± 2.5 |  |
| F2 | 0.5 | 0.25 | 212.3 ± 3.7 | 0.28 ± 0.01 | –13.7 ± 1.1 | 72.4 ± 2.2 |  |
| **F3 (Optimized)** | **0.5** | **0.5** | **150.5 ± 2.8** | **0.20 ± 0.01** | **−18.5 ± 0.59** | **90.2 ± 2.35** | **Optimal formulation** |
| F4 | 0.5 | 1.0 | 192.4 ± 3.2 | 0.24 ± 0.02 | –19.2 ± 1.3 | 82.6 ± 2.0 |  |
| F5 | 1.0 | 0.5 | 210.5 ± 3.1 | 0.27 ± 0.01 | –16.4 ± 0.85 | 79.3 ± 2.4 |  |
| F6 | 1.5 | 0.5 | 260.2 ± 3.9 | 0.30 ± 0.02 | –14.6 ± 0.69 | 75.1 ± 2.6 |  |
| F7 | 0.5 | 0 | 328.1 ± 5.6 | 0.39 ± 0.03 | –9.5 ± 0.7 | 60.5 ± 2.8 |  |

#### **Formulation Optimization Strategy**

To optimize the hybrid nanoparticle formulation, we systematically evaluated the effects of two critical formulation variables: **cholesterol concentration** (0.2–1.5 mM) and **Pluronic F68 content** (0–1.0% w/v). These parameters were selected based on their known impact on nanoparticle stability, particle size, and drug encapsulation efficiency (EE%), as reported in the literature. Based on literature survey and preliminary experiments, a total of seven formulations (F1–F7) were prepared by varying the concentrations of cholesterol and Pluronic F68 while keeping the curcumin:β-cyclodextrin complex ratio constant (1:1 molar ratio). The formulations were characterized for particle size, polydispersity index (PDI), zeta potential, and entrapment efficiency.

#### **Results and Discussion**

To optimize the hybrid nanoparticle formulation, we systematically investigated the impact of varying cholesterol (0.2–1.5 mM) and Pluronic F68 (0–1.0% w/v) concentrations on particle size, polydispersity index (PDI), zeta potential, and entrapment efficiency (EE%). A clear trend was observed where increasing cholesterol concentration beyond 0.5 mM led to a progressive increase in particle size and PDI, indicating compromised structural integrity, while formulations with 0.5 mM cholesterol maintained compact and stable nanoscale characteristics. Similarly, the incorporation of Pluronic F68 significantly influenced the colloidal stability and homogeneity of the particles. Absence of the surfactant led to poor stability and high PDI, whereas a 0.5% concentration of Pluronic F68 resulted in the most uniform and stable formulation (PDI = 0.20), with the highest EE% (90.2%) and optimal zeta potential (- 18.5 mV). Thus, formulation F3 (cholesterol 0.5 mM, Pluronic F68 0.5% w/v) was identified as optimal, balancing particle size (150.5 ± 2.8 nm), stability, and drug-loading capacity. These findings underscore the importance of precise component ratios in achieving reproducible and efficient nanoparticle delivery systems.
